# *In vivo* imaging of adeno-associated viral vector labelled retinal ganglion cells

**DOI:** 10.1038/s41598-018-19969-9

**Published:** 2018-01-24

**Authors:** Corey A. Smith, Balwantray C. Chauhan

**Affiliations:** 1 Department of Physiology and Biophysics, Dalhousie University, 5850 College Street, PO Box 15000, Halifax, Nova Scotia B3H 4R2 Canada; 2 Retina and Optic Nerve Research Laboratory, Dalhousie University, 5850 College Street, PO Box 15000, Halifax, Nova Scotia B3H 4R2 Canada; 3 Department of Ophthalmology and Visual Sciences, Dalhousie University, 1276 South Park Street, 2W Victoria, Halifax, Nova Scotia B3H 2Y9 Canada

## Abstract

A defining characteristic of optic neuropathies, such as glaucoma, is progressive loss of retinal ganglion cells (RGCs). Current clinical tests only provide weak surrogates of RGC loss, but the possibility of optically visualizing RGCs and quantifying their rate of loss could represent a radical advance in the management of optic neuropathies. In this study we injected two different adeno-associated viral (AAV) vector serotypes in the vitreous to enable green fluorescent protein (GFP) labelling of RGCs in wild-type mice for *in vivo* and non-invasive imaging. GFP-labelled cells were detected by confocal scanning laser ophthalmoscopy 1-week post-injection and plateaued in density at 4 weeks. Immunohistochemical analysis 5-weeks post-injection revealed labelling specificity to RGCs to be significantly higher with the AAV2-DCX-GFP vector compared to the AAV2-CAG-GFP vector. There were no adverse functional or structural effects of the labelling method as determined with electroretinography and optical coherence tomography, respectively. The RGC-specific positive and negative scotopic threshold responses had similar amplitudes between control and experimental eyes, while inner retinal thickness was also unchanged after injection. As a positive control experiment, optic nerve transection resulted in a progressive loss of labelled RGCs. AAV vectors provide strong and long-lasting GFP labelling of RGCs without detectable adverse effects.

## Introduction

The retina has served as a successful model for numerous significant advances in neuroscience. Because it is the only part of the central nervous system that can be optically imaged, the retina provides a unique opportunity to image and track retinal and optic nerve structures *in vivo* for both clinical and experimental diseases. Retinal ganglion cells (RGCs) are the output neurons of the retina whose axons provide the essential pathway for visual signals to reach the brain. A hallmark of glaucoma, one of the most common causes of irreversible visual disability and blindness, is progressive loss of RGC somas and axons. There are currently no methods of directly imaging living RGCs in humans to assess disease severity, instead measures of optic nerve head neuroretinal rim, retinal nerve fiber layer and ganglion cell layer thickness with modern imaging techniques, such as optical coherence tomography (OCT), are used as surrogates of how many RGCs axons and somas are present. However, the sensitivity and specificity of these surrogate measures of RGC degeneration, and ultimately death, is poorly understood^1,2^. It is also important to consider a proportion of RGC loss may not be due to disease, but rather normal ageing^3^. The ability to image RGC axons or somas directly would allow earlier and accurate detection of diseases such as glaucoma and with serial imaging, a more accurate rate of RGC loss as an indicator of disease progression.

Advances in adaptive optics imaging have allowed *in vivo* imaging of photoreceptors, the most abundant neuron in the retina, in monkeys and humans^4,5^. Unlike photoreceptors, which contain pigment, RGCs are transparent and therefore less amenable to imaging without the introduction of contrast material. Furthermore, because the ganglion cell layer contains other cell types, particularly, displaced amacrine cells^6^, the specificity of imaging RGCs in the ganglion cell layer is problematic without a RGC-specific indicator^7^. An indicator that is highly specific, reproducible and allows RGC quantification would represent a considerable advance in the assessment of glaucomatous damage.

Ideally, a clinically applicable method of labelling and imaging RGCs should be minimally invasive, have single cell resolution and have persistence such that longitudinal changes in RGC counts can be monitored^8^. One such method utilizes apoptotic indicators, annexin V^9^ or effector caspases^10,11^, to measure cell death in the retina of rodents and humans^12^ in conjunction with *in vivo* fluorescence imaging. The specificity of this approach for RGCs is not known and could lead to a high number of false positives when other retinal neurons are labelled. Furthermore, this approach provides evidence of cells undergoing cell death at a single time point, making it difficult to ascertain how many RGC are lost and how many remain, thereby making it challenging to document disease progression. Other investigators used genetically encoded calcium indicators for repeated *in vivo* functional imaging of foveal RGCs in macaques^13,14^. This method required implementation of adaptive optics to obtain sufficient fluorescence intensity in a small region of the retina, but represents important progress as it measures not only the presence of RGCs, but their functional responses to light stimuli via fluorescence intensity.

Adeno-associated viral (AAV) vectors have been used in clinical trials for retinal diseases with successful safety and transduction outcomes^15^. AAV vectors can be customized to improve cell-type specificity and rate of labelling. Such approaches include manipulation of the capsid (i.e., wild-type or engineered), genome (i.e., promoters and reporter gene, such as a fluorophore, e.g., green fluorescence protein (GFP)) and route of delivery (i.e., intravitreal or subretinal). Most recently, research has addressed the limited capacity for DNA, approximately 4.7 kilobases, in AAV vector mediated transduction. Small gene promoters of human DNA (“MiniPromoters”) were developed to drive gene expression in neural tissue^16,17^. One of these MiniPromoters is for the gene *DCX* that encodes for the protein doublecortin; previously shown to be expressed in developing and mature retinal neurons. Specifically, there is evidence the protein is present in RGCs as well as amacrine, bipolar and horizontal cells^18,19^. When the MiniPromoter developed for *DCX* was used in an AAV vector, it primarily targeted the RGCs in mice^16^. However, the efficiency, specificity and persistence of these promoters when incorporated into an AAV vector are not known. These tools provide an opportunity for *in vivo* labelling of RGCs with improved specificity.

In this study, we demonstrate the feasibility of AAV delivery via intravitreal injection as a means for *in vivo* RGC labelling in mice. This method was used to test if AAV vectors provide specific fluorescence labelling for longitudinal *in vivo* imaging. Confocal scanning laser ophthalmoscope (CSLO) imaging was used for *in vivo* detection of fluorescence labelling, while OCT imaging and electroretinography (ERG) was used to detect any structural or functional changes to the retina, respectively. It is expected the results could provide a minimally invasive method for efficient and robust RGC labelling with longitudinal imaging, offering the ability to detect changes in RGCs and track progression of diseases such as glaucoma.

## Results

**Figure 1:**
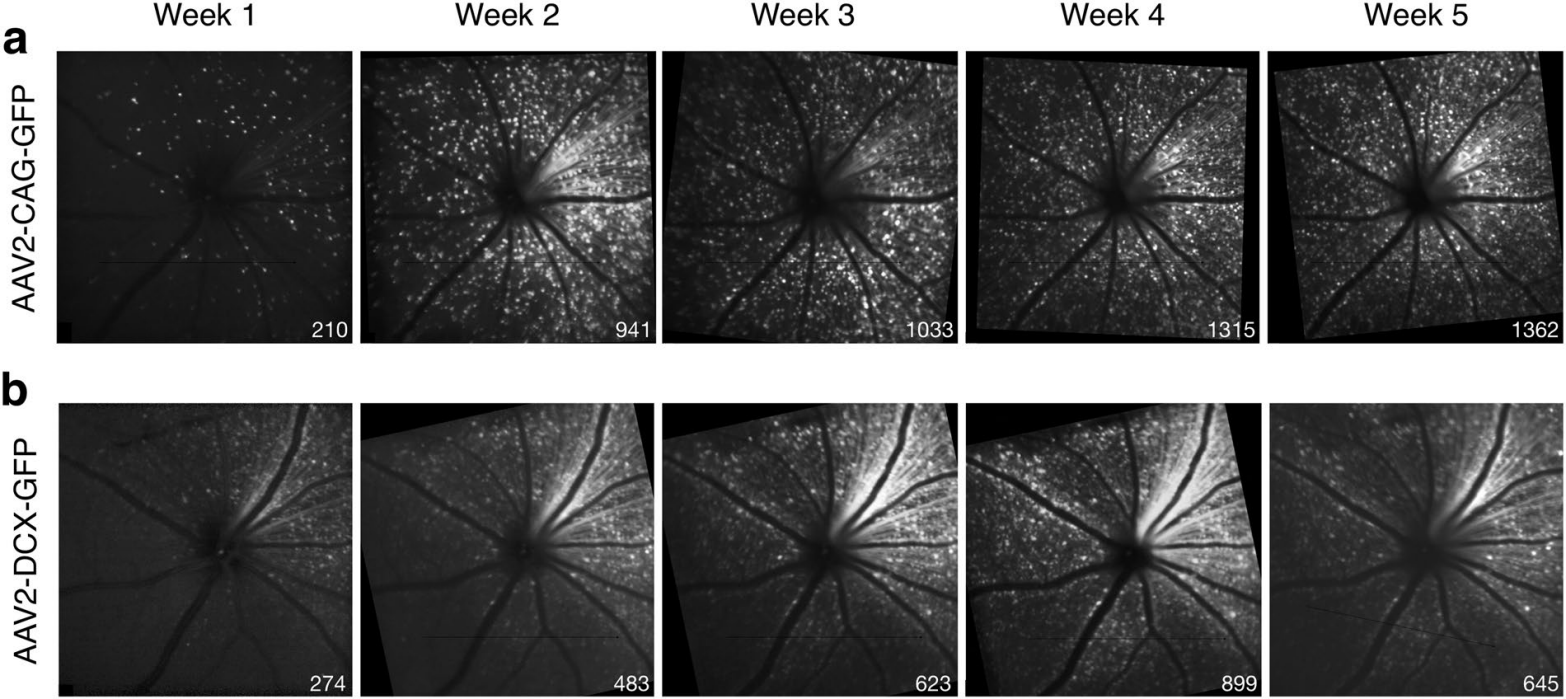
Longitudinal *in vivo* CSLO images of a mouse retina post-injection of AAV vectors. Fluorescence confocal scanning laser ophthalmoscopy (CSLO) images from the same animal and acquired sequentially up to 5-weeks following intravitreal injection of (**a**) AAV2-CAG-GFP and (**b**) AAV2-DCX-GFP. Images were acquired with a 488 nm excitation laser. Cell counts are shown for each fluorescence image (bottom right corner).

**Figure 2:**
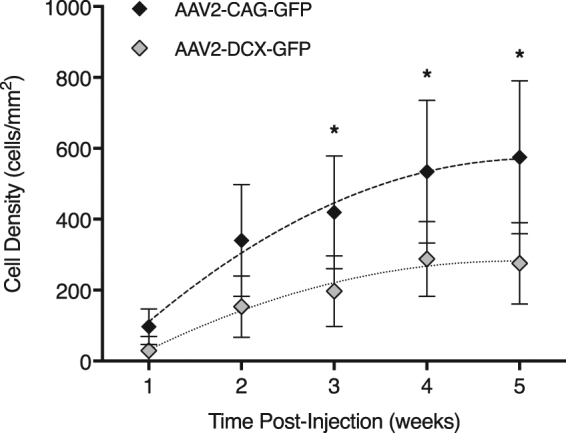
Comparison of *in vivo* GFP expression in the ganglion cell layer between vectors after intravitreal injection. Cell densities were calculated from 30-degree field of view (approximately 1.36 mm) fluorescence confocal scanning laser ophthalmoscopy images centred at the optic nerve head for the AAV2-CAG and AAV2-DCX vectors. Trendlines show the second-order polynomial regression results calculated for each group. Error bars represent 95% confidence intervals; *p < 0.05; n = 6.

Following intravitreal injection of the AAV vector, GFP labelling was detectable by *in vivo* CSLO fluorescence imaging in 17 mice (81%) at week 1 post-injection. The four (19%) animals that did not show any GFP labelling at week 1 did have labelling by week 2. Examples of the images acquired weekly are shown in two different animals labelled by vectors containing the *CAG* and *DCX* promoters (Fig. 1). Cells that were labelled in the early weeks post-injection continued to express the GFP until at least week 5. However, GFP labelling in the majority of cells was not evident until 2–4 weeks post-injection (Fig. 1). The *in vivo* mean (95% confidence interval) central retina cell density for the AAV2-CAG vector was 97 (50) cells/mm^2^ at week-1 and 534 (201) cells/mm^2^ at week-4. For the AAV2-DCX vector, cell density was 29 (40) cells/mm^2^ at week-1 and 288 (105) cells/mm^2^ at week-4. That is 77 (6)% of cells ultimately labelled with GFP at week 5, became GFP positive between weeks 1 and 5 in the AAV2-CAG injected animals and 85 (5)% of cells in the AAV2-DCX injected animals. The density of GFP labelled cells for both vectors follow a similar trend over time and fit with second-order polynomial models of *R*^2^ = 0.43 for AAV2-CAG and *R*^2^ = 0.38 for AAV2-DCX (Fig. 2). The AAV2-CAG vector labelled significantly more cells than the AAV2-DCX vector (*p* < 0.05), however, this difference was not apparent until week 3. At all subsequent time points, the percent difference of labelled cells between the AAV2-CAG and AAV2-DCX vectors was an average of 65 (10)% (Fig. 2). The trend of increasingly more labelled cells over time occurred until approximately week 4 post-injection, at which time neither vector had significantly more cells labelled than the previous week consecutively for two time points. The proportion of retina labelled in the *in vivo* images with the AAV2-CAG vector was 97 (7)% whereas the AAV2-DCX was 53 (30)%.

Both the AAV2-CAG and AAV2-DCX vectors had a high signal-to-background ratio (Table 1) and difference in means equal to 0.67 (0.90). The measures of image quality were also comparable between the two vectors for signal-to-noise ratio and contrast-to-noise ratio with percent differences of 9.7% and 3.3%, respectively. There was no statistically significant difference between the vectors for any of the measures (Table 1).

**Table 1:** Signal intensity and image quality measures of retinal ganglion cell labelling with the two AAV vectors.

	Signal-to-Background		Signal-to-Noise		Contrast-to-Noise	
	Ratio	*p*	Ratio	*p*	Ratio	*p*
AAV2-CAG	2.97 (0.66)	0.08	6.84 (1.35)	0.49	4.35 (0.49)	0.85
AAV2-DCX	2.30 (0.33)		7.54 (1.30)		4.21 (1.09)	

Signal-to-background ratio measured the intensity ratio between the signal and background, signal-to-noise ratio and contrast-to-noise ratio represents the image quality at 5-weeks post-injection. Ratios are expressed as mean (95% CI). No statistically significant difference was found for any measure.

**Figure 3:**
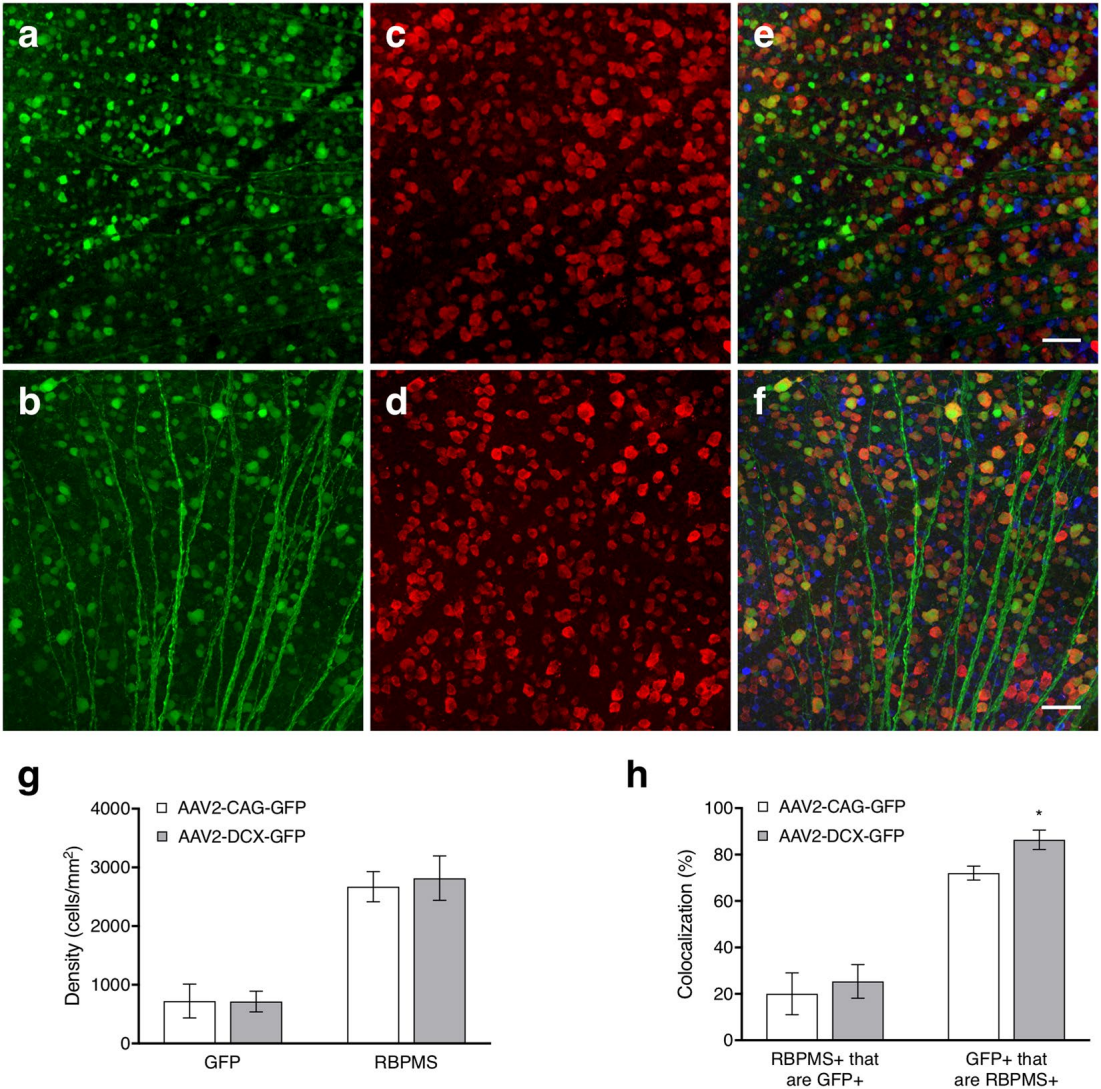
Cellular labelling in flat-mount retinas with the two AAV2 vectors. Panels (a,c and e) are micrographs from an animal that received an injection of the AAV2-CAG-GFP vector while panels (b,d and f) are from an animal that received an injection of the AAV2-DCX-GFP vector. (**a**,**b**) Show GFP labelling via intravitreal injection of AAV2-GFP vector (*green*); (**c**,**d**) immunohistochemical labelling of RGCs with RNA binding protein with multiple splicing (RBPMS, *red*) and (**e**,**f**) respective merged images with choline acetyltranferase (ChAT, *blue*). Scale bars = 50 μm. (**g**) Density of GFP positive cells from an AAV intravitreal injection and RBPMS positive cells from immunohistochemical labelling. (**h**) RBPMS+ cells that are GFP+ indicates the proportion of RGCs labelled by GFP and GFP+ cells that are RBPMS+ indicates the specificity of the AAV vector to RGCs. Histograms show means and error bars show 95% confidence intervals; *p < 0.05; n = 6.

Figure 3 shows example micrographs with labelling from intravitreal injections (panels a-b) and immunohistochemical markers (panels c-f). The co-localization of GFP positive cells in the ganglion cell layer with RBPMS are shown in the merged micrographs in addition to the distribution of cholinergic amacrine cells (Fig. 3e,f). The cell densities of GFP labelled cells, 723 (287) cells/mm^2^ for AAV2-CAG and 715 (177) cells/mm^2^ for AAV2-DCX, revealed the AAV labelling via intravitreal injection was not significantly different between the two vectors (Fig. 3g, *p* = 0.97). Based on immunohistochemical labelling, RGC densities of 2671 (256) cells/mm^2^ for AAV2-CAG and 2817 (379) cells/mm^2^ for AAV2-DCX were not significantly different *(p* = 0.55). Co-localization showed the specificity of GFP labelling to RGCs to be very high at week 5 post-injection for both vectors (Fig. 3h) with the proportion of GFP positive cells that were RGCs as 72 (3)% for the AAV2-CAG vector and 86 (4)% for the AAV2-DCX vector. There was significantly higher specificity of RGC labelling with the AAV2-DCX vector (*p* < 0.05) and it was independent of the region of retina (central vs. peripheral). However, the proportion of RGCs labelled by each vector (RBPMS+ that are GFP+) was 20 (9)% for AAV2-CAG and 35 (7)% for AAV2-DCX and not significantly different between vectors (*p* = 0.38). For the AAV2-DCX group, a paired t-test showed that there was a significant difference between the *in vivo* and *ex vivo* densities, 242 (111) cells/mm^2^ and 716 (177) cells/mm^2^, respectively; *p* < 0.05. Linear regression revealed *R*^2^ = 0.01 for *in vivo* vs. *ex vivo* GFP densities and *R*^2^ = 0.16 for *in vivo* GFP density and *ex vivo* RBPMS density.

**Figure 4:**
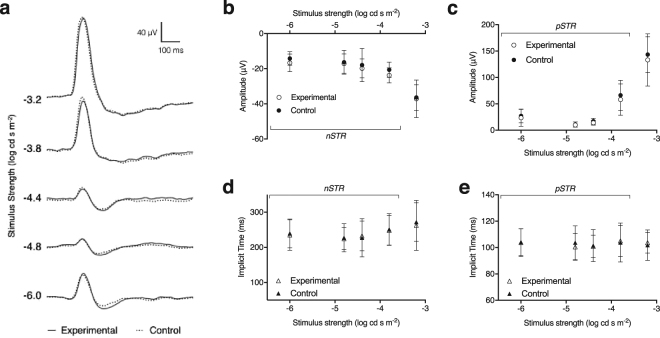
Effects of AAV2-DCX-GFP injection on the ERG 5-weeks post-injection. (**a**) Example waveforms of ERG recordings obtained from AAV injected experimental (*solid*) and control fellow (*dashed*) eyes over a range of low stimulus strengths. Averaged group data for control (*filled circles*) and experimental (*unfilled circles*) of (**b**) negative STR, (**c**) positive STR amplitudes. Averaged group data for control (*filled triangles*) and experimental (*unfilled triangles*) eyes of (**d**) negative STR implicit times, and (**e**) positive STR implicit times. No significant differences were found between the control and experimental eyes for any of the measures. Error bars represent 95% confidence interval; n = 6.

At baseline, prior to the intravitreal injection, there was no significant difference of the amplitude or implicit time between left and right eyes (Supplementary Fig. S1). Week 5 post-injection there was no significant decrease in the positive (*p* = 0.44) and negative (*p* = 0.84) STR amplitudes or implicit times (pSTR, *p* = 0.31 and nSTR, *p* = 0.33) between the control and experimental eyes following intravitreal injections with the AAV2-DCX vector, indicating the RGC contribution to the ERG was unaffected (Fig. 4). The mean relative pSTR amplitude was 0.86 (0.04) and nSTR amplitude was 1.12 (0.06) across all signal strengths. Furthermore, the amplitudes of the a-wave, 187 (26) µV vs. 184 (23) µV, *p* = 0.87, and b-wave, 332 (108) µV vs. 315 (80) µV, *p* = 0.87, were not significantly different between the experimental and control eyes, respectively. The ERG response in the animals that received an optic nerve transection demonstrated significantly reduced pSTR and nSTR amplitudes, with the pSTR amplitude in the transected eye 47 (9)% that of control eye, while the nSTR amplitude was 83 (14)% that of the control eye (Supplementary Fig. S2).

**Figure 5:**
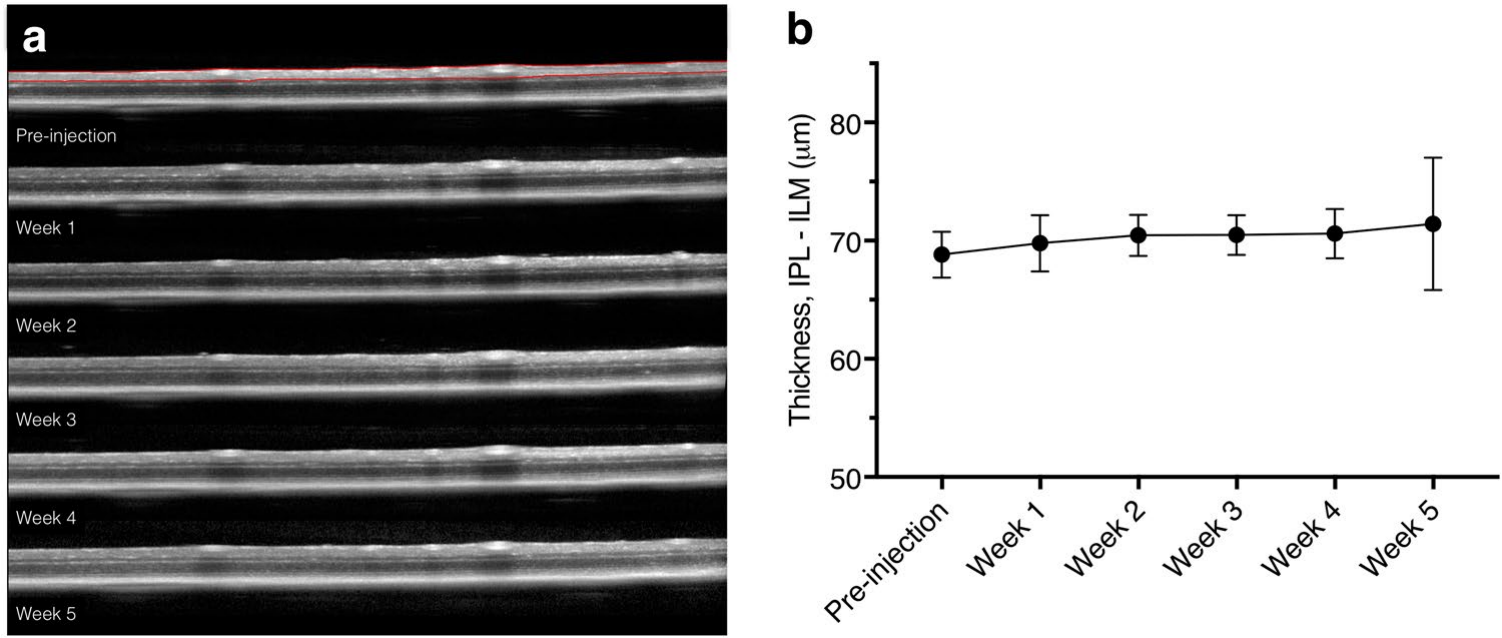
Longitudinal optical coherence tomography (OCT) scans of an eye following AAV2-DCX-GFP injection. (**a**) Representative OCT scans in one animal obtained from the raster scanning pattern at pre-injection and at weeks 1, 2, 3, 4 and 5 after intravitreal injection. Red lines in pre-injection scan show segmentation of the inner retina (from inner plexiform layer to inner limiting membrane). (**b**) Mean inner retinal thickness measurement for the injected eyes measured longitudinally. No statistically significant differences were found between any of the time points. Error bars represent 95% confidence interval; n = 10.

Inner retinal thickness, the region of the retina between the ILM and IPL, was used to include the ganglion cell layer and retinal nerve fibre layer (Fig. 5a). The range of mean thicknesses in all animals across all time points was 65–72 µm. The mean proportional change in ILM-IPL thickness compared to pre-injection was 1.025 (0.007) and no significant change (*p* = 0.51) in the thickness between time points was shown up to five weeks after an intravitreal injection of an AAV vector (Fig. 5b).

**Figure 6:**
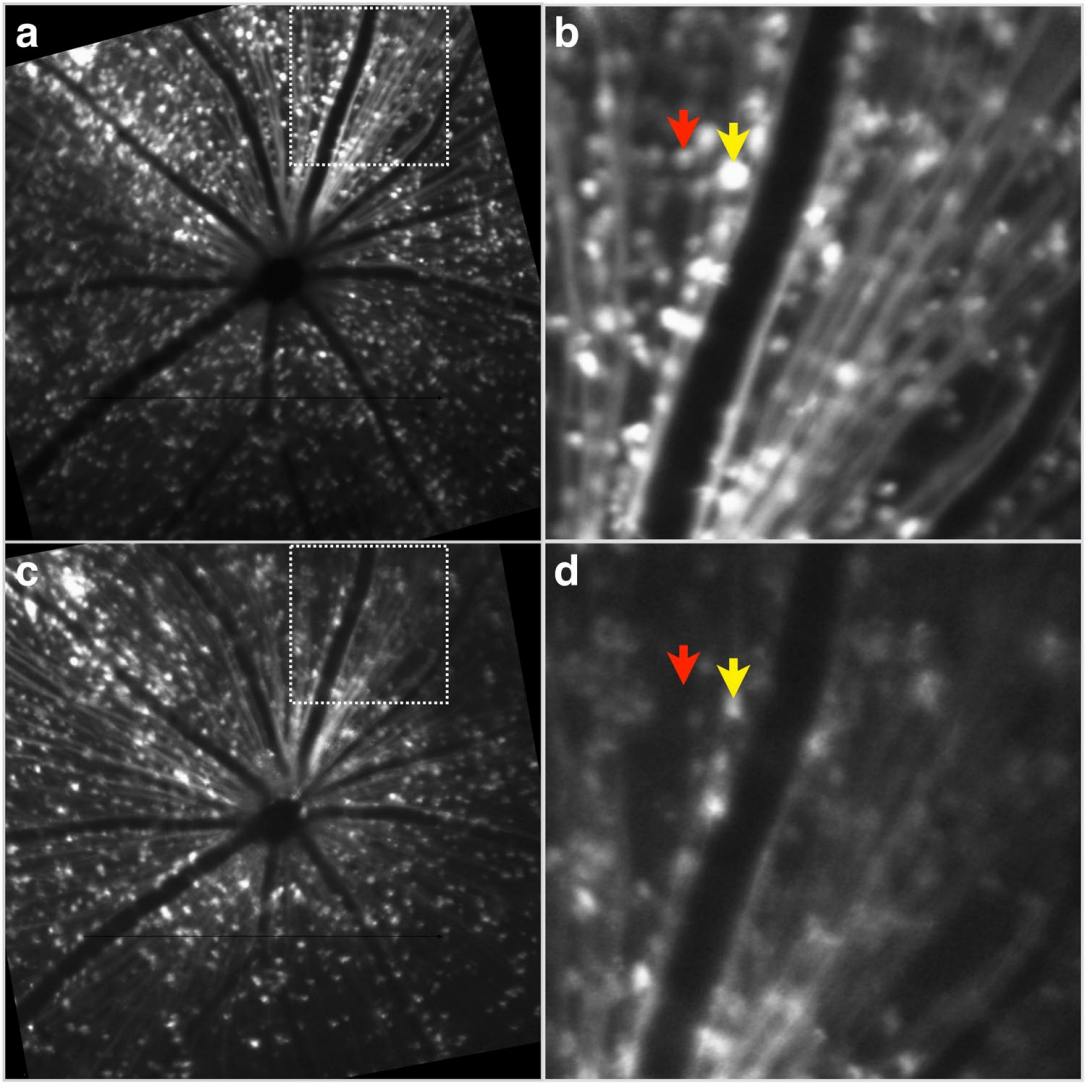
GFP labelling before and after optic nerve transection. *In vivo* fluorescence CLSO images from the same animal and retinal region, but at different time points, following optic nerve transection. (**a**,**b**) 5 weeks post-injection of the AAV2-DCX-GFP vector to label RGCs and (**c**,**d**) 14 days post-optic nerve transection to demonstrate the effects of RGC death on GFP labelling. Panels b and d are higher magnification views of the respective regions marked by boxes in panels a and c. Arrows indicate examples of a cell that has lost all fluorescence (*red*) and a cell with diminished fluorescence (*yellow*).

Longitudinal fluorescence images corresponding to the same region of the retina before and after optic nerve transection are shown in Fig. 6. The *in vivo* AAV2-DCX-GFP labelling is clearly visible, but at 14 days post-transection there was a 23 (7)% decrease in GFP labelled cells with some cells losing all fluorescence (red arrow, Fig. 6), while others had diminished fluorescence (yellow arrow, Fig. 6).

## Discussion

In this study we demonstrated that intravitreal injection administration of AAV vectors with a fluorescent reporter gene provides robust and sustained *in vivo* RGC labelling in mice. The GFP labelling was sufficiently strong to be detectable by non-invasive CSLO imaging with laser sources approved and used routinely in clinical practice. Between week 2 and week 4 there was a 69% increase in labelling for AAV2-CAG and 80% for AAV2-DCX. Furthermore, the GFP signal was persistent in individual RGCs for at least five weeks (Fig. 1). There are two conclusions from the fluorescence trend important for the planning of longitudinal RGC survival experiments. Firstly, since the transduction of AAV vector requires approximately four weeks for maximal labelling (Fig. 2), consistent with previous studies^20,21^, it was notable that GFP labelling was cumulative during this time and that the fluorescence signal was not transient. Secondly, we demonstrated a progressive decrease in RGC density following optic nerve transection (Fig. 6). Furthermore, the *in vivo* electrophysiological assay of RGC health showed the AAV intravitreal injection did not cause measureable damage (Fig. 4). These findings demonstrate that our technique of RGC labelling is a viable method to monitor real-time RGC survival in experimental disease models and interventions for neuroprotection and neuroregeneration.

AAV vector serotypes and capsids show preferential transduction to differing retinal cell types, even with ubiquitous promoters^21–23^, and is dependent on the injection site (e.g., subretinal vs. intravitreal). Serotype 2 was chosen for this study because it most often shows the best transduction efficiency in RGCs when administered by intravitreal injection^24,25^. Serotypes and engineered capsids^23^ can be used for improving transduction efficiency of cells, while promoters can be used for improving transduction specificity. Ubiquitous promoters are commonly used, even when targeting specific cells, but could drive protein synthesis and cause side effects in other tissues and cells^26,27^. Therefore, use of specific promoters such as *DCX*, helps provide improved cell specific targeting and reduce the possible side effects induced by ubiquitous promoters. The specificity of RGC labelling with AAV vectors or comparing promoters has not been previously quantified with RGC-specific immunohistochemistry or markers. Transduction efficiency in the retina was either measured by total density of labelled cells across the retina^28^ or fluorescence intensity within the retinal layers^29^. We demonstrated a 19% increase in specificity to RGCs by utilizing a specific promoter compared to a ubiquitous promoter while labelling approximately the same proportion of total RGCs (Fig. 3h). Choline acetyltransferase antibody labelling was used to show the presence of amacrine cells in the ganglion cell layer and did not co-localize with GFP positive cells. These results, in processed retinal tissue, demonstrated that the method of delivering fluorescent proteins via AAV2 and the specific *DCX* promoter yields high specificity for RGC labelling. A higher density of labelling was detected *ex vivo* than *in vivo* with the imaging methods we chose, though due to the small sample size in this study, we cannot determine the level of correlation between *in vivo* and *ex vivo* cell densities. *Ex vivo* tissue microscopy allowed for higher resolution imaging than the CSLO system and could have resulted in a larger number of detectable cells. Alternatively, further work could determine if AAV transduction/labelling is representative of the entire RGC population or is preferential to a specific RGC type.

We previously showed that intravitreal injection of the neuronal tracer, cholera toxin subunit B (CTB), resulted in fluorescence labelling of cells in the ganglion cell layer detectable by *in vivo* imaging for at least 100 days post-injection. However, the CTB labelling was not specific to RGCs, with only 53% of CTB labelled cells being RGCs^30^. Other researchers have described AAV-mediated GFP gene transfer for cross-sectional^28,31^ or longitudinal^32^ fluorescence imaging of the retina, but there is no previously published work that has quantified RGC density from *in vivo* images or reported the specificity of GFP labelling to RGCs. Martin *et al*. estimated a 75–85% transduction of RGCs in rats, approximately 2.5 times higher compared to our findings in mice, with AAV vectors containing GFP under the control of the ubiquitous CBA promoter and modified to include the woodchuck hepatitis post-transcriptional regulatory element (WPRE)^28^. However, the high rate of transduction was achieved within 2 weeks after intravitreal injection and was attributed to the use of WPRE. The strengths of the RGC labelling studies described above, AAV vector labelling and specificity quantification, were combined to develop the protocol described in this study. The results imply that *in vivo* imaging of labelled RGCs from AAV vectors can provide a reliable method to quantify RGCs repeatedly and longitudinally. This is an improvement on widely used transgenic animals or invasive labelling techniques, such as retrograde labelling via the superior colliculus, as neither are clinically applicable methods for visualizing RGCs.

While the technique of RGC labelling described in this study does not label the entire RGC population, it does adequately sample the total RGC population. The reported *in vivo* density measures provide an estimate of labelling across the retina with signal intensity that was at least twice that of the background. This is an indication that the labelled cells are dispersed such that the resolution of the imaging system is able to adequately resolve individual cells. If all RGCs were transduced, it would be challenging to differentiate and quantify RGCs *in vivo* due to the high density labelling. In some applications of RGC targeting, such as high-resolution imaging and neuroprotective applications, it could be beneficial to have higher transduction. The inner limiting membrane (ILM) has been reported to be a barrier to viral transduction via intravitreal delivery^33^. This is likely the explanation for the observed cases of uneven labelling across the retina and a higher degree of GFP in the area closest to the injection site (Supplementary Figure S3). A higher density of RGC labelling could have been achieved with improved transduction by compromising the ILM. To avoid possible disruption or integrity of the retina, we chose not use strategies such as enzymatic degradation of extracellular matrix of the ILM^33,34^, vitreous aspiration prior to intravitreal injection^35,36^, formation of a bleb below the ILM to create a space for vector bolus^37^, or ILM peeling^38^, to reduce the resistance of the ILM to AAV transduction.

In summary, this work provides a novel method for quantifying RGCs in experimental studies and monitoring RGC loss in animal models. The results show that a minimally invasive intravitreal injection of AAV vectors reliably labels RGCs in mice for longitudinal *in vivo* visualization (Fig. 1), *in vivo* quantification (Fig. 2), *ex vivo* labelling (Fig. 3), and *ex vivo* quantification (Fig. 3g). We show that the measures of RGC structure (retinal thickness) and function (ERG measures) are not affected by the administration of the AAV vectors. The high specificity of these AAV vectors to RGCs indicates there is potential for diagnostic and therapeutic applications in diseases that cause RGC loss, such as glaucoma. Specifically, it would reduce the challenges presented by a heterogeneous population of cell types in the ganglion cell layer and the large amount of variability in the number of RGCs between individuals. Future work is required to investigate if AAV vectors can efficiently, reliably and specifically transduce human RGCs and the effects of introducing exogenous reporters into human RGCs. Virus-based strategies are used for therapeutics, but their use for diagnostics would represent a paradigm shift and major advance in the management of ocular neuropathies.

## Methods

### Study Approval

Animal procedures complied with the Canadian Council of Animal Care standards and animal ethics approval was obtained from the University Committee on Laboratory Animals at Dalhousie University.

### Adeno-Associated Viral Vector

Two different AAV2 serotype recombinant vector constructs were employed. The first utilized the cytomegalovirus early enhancer/chicken β actin (CAG) promoter to drive the expression and synthesis of the reporter, enhanced GFP, packaged in recombinant AAV2 wildtype capsid (AAV2-CAG-GFP, Vector Biolabs, Malvern, PA, USA). The second utilized a tissue or cell specific promoter from the *DCX* gene to drive the expression and synthesis of the reporter, humanized GFP, packaged in recombinant AAV2 capsid with quadruple tyrosine residues mutated to phenylalanine (AAV2-DCX-GFP, provided by Dr. William Hauswirth, Retinal Gene Therapy Group, University of Florida, Gainesville, FL, USA).

### Animals

Adult female C57BL/6 mice (JAX™ Mice Stock Number: 000664, Charles River Laboratories, Saint-Constant, QC, Canada) were used. Mice were housed in a 12-hour light-dark cycle and provided food and water *ad libitum*. Mice were divided into the following groups: group 1 for AAV2-CAG-GFP labelling (n = 6), group 2 for AAV2-DCX-GFP labelling (n = 7), group 3 for ERGs and group 4 for optic nerve transection following intravitreal injection of AAV2-CAG-GFP or AAV-DCX-GFP (n = 2).

### Intravitreal Injection

Mice were anaesthetized with a mixture of ketamine (100 mg/kg body weight) and xylazine (10 mg/kg body weight). The left eye was dilated topically with one drop of 1% tropicamide (Alcon Canada Inc., Mississauga, ON, Canada) and continuously rehydrated with lubricant eye drops. Under an operating microscope, a scleral puncture approximately 0.5 mm from the limbus was made with a 30 G hypodermic needle. A 33 G needle (Hamilton Company, Reno, NV, USA) was then inserted into the puncture and 1.5 µl of the vector injected slowly into the vitreous cavity with at titer of least 1 × 10^12^ vg μl^−1^ for the *CAG* promoter and 3.94 × 10^12^ vg μl^−1^ for the *DCX* promoter. After the injection, antibacterial drops and ophthalmic gel (Novartis Pharmaceuticals Canada Inc.) were applied.

### *In vivo* Imaging

*In vivo* imaging was performed with a confocal scanning laser ophthalmoscope (CSLO) and spectral domain optical coherence tomography (OCT) device modified for use in mice (Spectralis Multiline, Heidelberg Engineering GmbH, Heidelberg, Germany)^39^. CSLO imaging was performed to visualize the GFP-positive cells after intravitreal injection while OCT imaging was used to derive B-scans of the retina to quantify inner retinal thickness (see below). All laser sources were Class 1 according to International Electrotechnical Commission (IEC)^40^.

The left pupil was dilated (one drop of 1% tropicamide and one drop of 2.5% phenylephrine hydrochloride, Alcon Canada) and the mouse anaesthetized with inhalant isoflurane, induction of 3–4% volume (Baxter Corporation, Mississauga, ON, Canada) with 1.5 L/min oxygen flow and maintained at 1.5–3% volume with 0.8 L/min oxygen flow, via a nose cone attached to a portable inhalation system. The mouse was placed on a heating pad for the duration of imaging. Ophthalmic gel and a custom-made polymethyl methacrylate plano contact lens (Cantor and Nissel Limited, Brackley, UK) were used to maintain corneal hydration and improve image quality. Baseline images focused at the level of the nerve fiber layer were first acquired with infrared (820 nm) illumination. Fluorescence images were then acquired in CSLO mode with a 488 nm excitation laser and bandpass filter of 505–545 nm. Each image was averaged a minimum of 20 times with automatic real-time eye tracking software to increase the signal-noise ratio. The imaging protocol was repeated at several time points post-injection (Supplementary Fig. S4) with the image tracking software to ensure that the same retinal areas were imaged in each session.

The same animal protocol and imaging set-up was used for OCT imaging to quantify inner retinal thickness. The principles of OCT for retinal imaging have been described elsewhere^41^; briefly, the technique employs the principle of low coherence interferometry to generate high-resolution cross-sectional images of the retina. A raster pattern of 19 equally spaced horizontal B-scans, each 30 degrees wide, centred on the optic nerve head was used (Supplementary Fig. S5). The scanning speed was 40 000 A-scans per second and each B-scan comprised of 1536 A-scans. Each B-scan was averaged 20 times.

### Electroretinography

Full field ERG analysis was performed prior to intravitreal injection and at week 5 post-injection to determine if the AAV-mediated GFP labelling driven by the *DCX* promoter had detrimental effects on retinal function (Supplementary Fig. S4). To demonstrate the ability of our ERG protocol to detect changes in retinal function, specifically that of the RGCs, a subset of animals received an optic nerve transection (Supplementary Fig. S2). Mice were dark-adapted overnight (≥12 hrs) before being anesthetized with an intraperitoneal injection of ketamine (100 mg/kg) and xylazine (10 mg/kg). Pupils were dilated with one drop each of 1% tropicamide and 2.5% phenylephrine hydrochloride (Alcon Canada Inc., Mississauga, ON, Canada). Body temperature was maintained between 35 and 37 °C with a heating pad and monitored with a rectal probe. A platinum subdermal electrode (Grass Instruments, Quincy, MA, USA) was placed in the base of the nose for reference. A microfiber electrode (DTL Plus Electrode**™**, Diagnosys, Littleton, MA, USA) was placed on the corneal surface of each eye with ophthalmic liquid gel (Tear-Gel, Novartis Pharmaceuticals Canada, Inc., Mississauga, ON, Canada) applied to maintain hydration and conductivity. Impedances of the corneal and ground electrodes were measured at 30 Hz and the ERG protocol was commenced only if impedance values were <150 ohms.

Signal amplification and recording setup are detailed elsewhere^42^, however briefly, flash stimuli were delivered with a Ganzfeld stimulator (LKC Technologies, Gaitherburg, MD, USA) and attenuated by neutral density filters (Kodak Wratten, Rochester, NY, USA). After a ten-minute stabilization period, ERG responses were recorded in the following order of flash intensity: −3.2, −3.8, −4.4, − 4.8, −6.0, −6.8 and 1 log cd s m^−2^. For flash intensities between 1.0 and −4.4 log cd s m^−2^, between 2 to 10 responses were averaged, while for the two lowest intensities, 10 to 14 responses were averaged. Each response was obtained with a 4.5 second interval between flashes.

### Optic Nerve Transection

Animals that underwent optic nerve transection were injected with AAV vector 5-weeks prior. Mice were anaesthetized using inhalant isoflurane as described for *in vivo* imaging. Under an operating microscope, the globe of the left eye was rotated downwards and held in place with a 9-0 conjunctival suture. To expose the optic nerve, an incision was made in the skin near the supraorbital ridge then the intraorbital subcutaneous tissues were dissected. The optic nerve dura was cut longitudinally and the optic nerve transected completely approximately 0.5 mm from the globe. The ophthalmic artery, located underneath the transected nerve, was kept intact. The incision was closed and the fundus examined to confirm no ischemic damage.

### Immunohistochemistry

To estimate the cell density and specificity of GFP labelling in the whole retina, immunohistochemistry was performed on retinal flat-mounts. Five weeks post-intravitreal injection animals were sacrificed with an overdose of sodium pentobarbital by intraperitoneal injection. The cornea and lens were removed and the eye-cups fixed in 4% paraformaldehyde for 2–3 hours. Retinas were washed in 1 × phosphate-buffered saline (PBS) for 10 min and incubated in blocking buffer (10% normal donkey serum, 0.3% Triton X-100) overnight at 4 °C. Retinas were incubated for 6 days at 4 °C in primary antibodies against RNA binding protein with multiple splicing^43–45^ (RBPMS, 1:1000 guinea pig anti-RBPMS, gift from Dr. Nicholas Brecha) and choline acetyltransferase (ChAT, 1:100 goat anti-ChAT, Millipore, Billerica, MA, USA) to identify RGCs and cholinergic amacrine cells, respectively. Retinas were then washed in PBS and incubated overnight at 4 °C in Alexa Fluor 488 (1:400 Alexa Fluor^®^ 488 conjugated rabbit anti-GFP, Molecular Probes, Eugene, OR, USA), Cy3 (1:1000, Cy3 conjugated donkey anti-guinea pig (Jackson Immuno Research Laboratories Inc., West Grove, PA, USA) and Alexa Fluor^®^ 633 (1:1000 Alexa Fluor^®^ 633 conjugated donkey anti-goat, Molecular Probes). Retinas were rinsed in PBS, mounted with anti-fade fluorescent mounting medium and coverslipped.

### Image Data Analysis

Image processing, analysis and cell quantification algorithm implementation of CSLO acquired images (1536 × 1536 pixels) were performed with a customized analysis tool in MATLAB software. Measures of signal intensity and image quality for *in vivo* fluorescence images were calculated (signal-to-background ratio, signal-to-noise ratio, and contrast-to-noise ratio) at week 5 post-injection. This was completed to determine if the GFP variants (enhanced vs. humanized) affected the signal intensity or image quality. For cell quantification a Gaussian filter (σ = 3, h = 19) was used to remove noise and a minima transform (h = 10) implemented to extract markers for each labelled cell. If a labelled region was greater than 200 pixels, it was assumed to be a cluster of cells and an eroding function applied as a method of segmentation. Connected components in the binary image were automatically counted and markers were superimposed on the original CSLO image to indicate their position. The final cell quantification was performed after manual correction of the automated algorithm to include cells not correctly identified, or to exclude objects incorrectly identified as cells. The total number of labelled cells divided by the retinal area, excluding the optic nerve region, was used to calculate cell density. A percentage of labelled retinal area in the 30° *in vivo* images was measured at week 5 post-injection by tracing and calculating the region with prominent labelling then divided by the total image area.

OCT layer segmentation was performed with the device segmentation algorithm (Heidelberg Eye Explorer, Heidelberg Engineering) after which each B-scan was checked for segmentation errors and manually corrected when required (Supplementary Fig. S5). The retinal nerve fibre and ganglion cell layers in mice are very thin, especially beyond the peripapillary region, and therefore cannot be reliably identified in OCT images. The signal interface between the inner plexiform layer and inner nuclear layer is easily identifiable and therefore used for segmentation. Inner retinal thickness was measured from the vitreous-retina surface to the inner plexiform layer (Supplementary Fig. S5).

Micrographs of retinal flat-mounts were imaged with a Zeiss Axio Imager M2 microscope (Carl Zeiss AG, Oberkochen, Germany) and a 20 × Plan-Apochromat objective (Carl Zeiss). Fluorescence images of the ganglion cell layer were captured with the ZEN software (Carl Zeiss) sampling the central, mid-peripheral, and peripheral retinal regions, with respect to the optic nerve head. In each sampled region, the number of cells labelled by (1) GFP, (2) RBPMS, and (3) RBPMS with GFP were quantified independently in graphics editing software (Adobe Photoshop CS6, Adobe Systems Incorporated, San Jose, CA, USA).

### ERG Data Analysis

ERG waveforms were analyzed with a custom toolbox for Matlab (Mathworks, Natick, MA, USA) and filtered with a low-pass eighth-order Butterworth filter at 50 Hz prior to measuring amplitudes. Analysis of the scotopic threshold response (STR) included amplitude measurement of the positive component (pSTR), from the baseline to the initial peak, and the negative component (nSTR), from baseline to the local minimum after the pSTR. Both the pSTR and nSTR signals have been shown to reliably measure RGC function in mice^42,46^. For photoreceptor response, the a-wave amplitude was measured from the baseline to the maximum negative trough, while the b-wave was measured from the a-wave trough to the maximum positive peak. For comparison between experimental and control eyes, the relative amplitude (experimental/control eye) was calculated.

### Statistics

Statistical analyses were performed in the open-source R platform (version 3.1.3, R Core Team, http://www.R-project.org) and Prism (version 7 for Mac, GraphPad Software, La Jolla, CA, USA). The Shapiro-Wilk normality test was used to test data for a normal distribution. Unless otherwise indicated, all results are expressed as mean (95% confidence interval) and statistical significance was assumed when *p* < 0.05. For *in vivo* cell densities second-order polynomial regression was used for each vector and the Holm-Sidak’s multiple comparisons test was used to test significance between vectors at each time point. Two-way analysis of variance (experimental/control vs. ERG stimulus strength) was applied to test the significance of the ERG data.

### Data Availability

The datasets generated during and/or analysed during the current study are available from the corresponding author on reasonable request.

## Electronic supplementary material

Supplementary Information
