# Supplementary material for: In vivo imaging of adeno-associated viral vector labelled retinal ganglion cells
Source: Sci Rep. 2018 Jan 24;8:1490. doi: 10.1038/s41598-018-19969-9 (PMC5784170; doi:10.1038/s41598-018-19969-9)
Supplement: Supplementary file 1 — Supplementary Information [file 41598_2018_19969_MOESM1_ESM.pdf]

Supplementary Information

In vivo imaging of adeno-associated viral vector labelled retinal ganglion cells

Corey A. Smith, Balwantray C. Chauhan

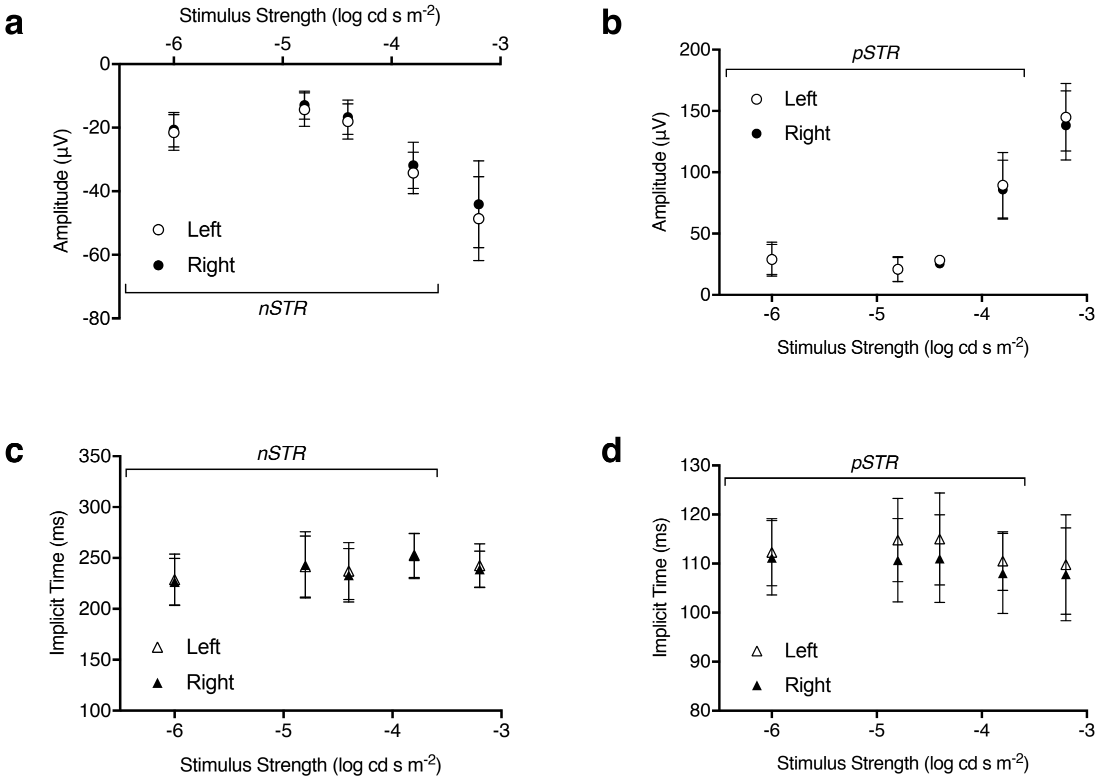

Supplementary Figure S1 Baseline ERG measures prior to intravitreal injection. Averaged group scotopic threshold responses (STR, within the bracketed stimulus strengths shown) for the right (*filled circle or triangle*) and left (*unfilled circle or triangle*) eyes for **a)** negative scotopic threshold response (STR) amplitudes, **b)** positive STR amplitudes, **c)** negative STR implicit times, and **d)** positive STR implicit times. No significant differences were found between the left and right eyes. Error bars represent 95% confidence intervals; n = 6.

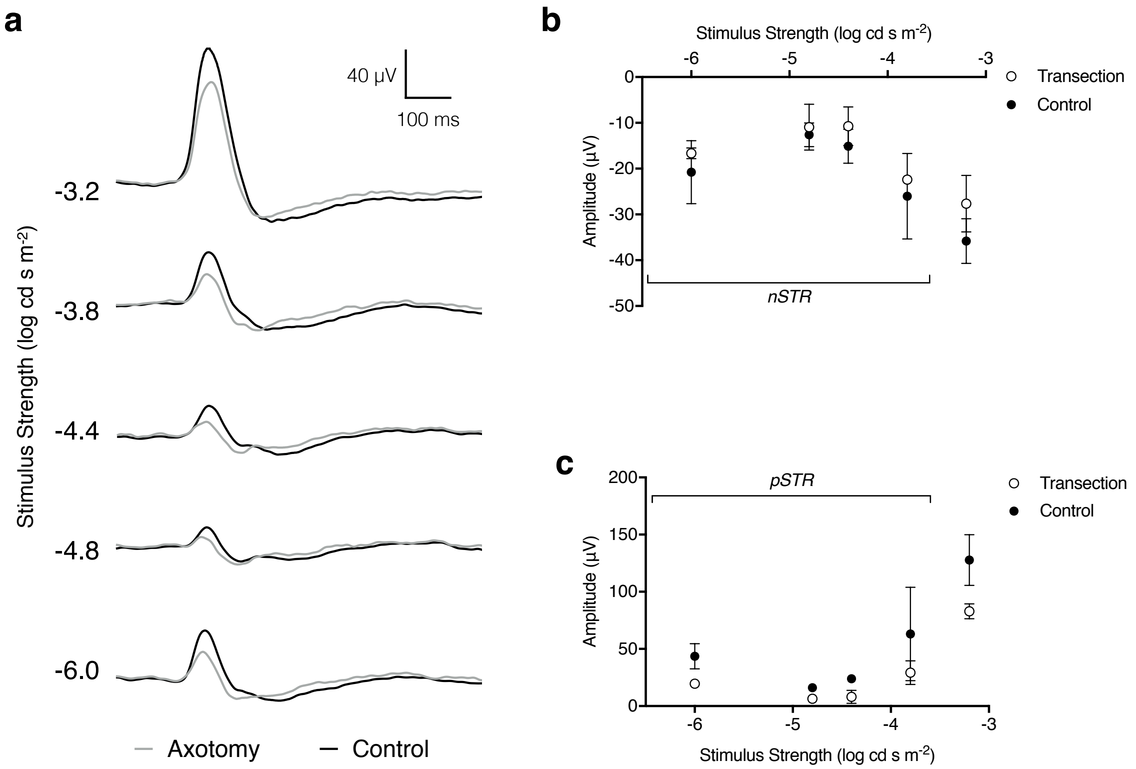

Supplementary Figure S2 Effects of optic nerve transection on the ERG. a) Example waveforms of ERG recordings obtained from control (*black*) and transected optic nerve (*gray*) eyes over a range of low stimulus strengths. Averaged group data for control (*filled circles*) and transection (*unfilled circles*) eyes of **b)** negative STR and **c)** positive STR amplitudes. Error bars represent 95% confidence interval; n = 2

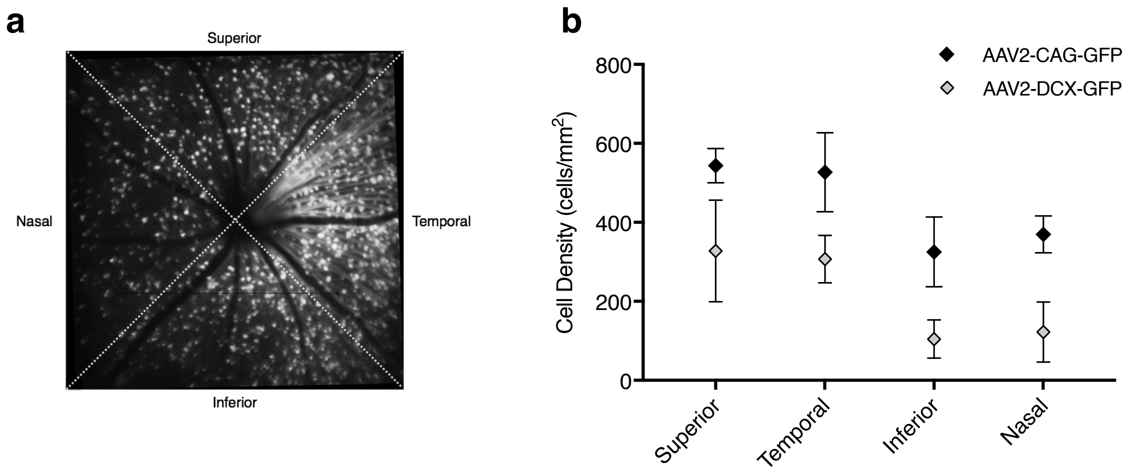

Supplementary Figure S3 Regional differences of labelling efficiency. Intravitreal administration of AAV vectors can result in preferential labelling near the site of injection. To determine whether there was an effect on labelling efficiency as a result of the superior-temporal injection site we examined **a)** four regions of each *in vivo* fluorescence image and **b)** quantified the cell density in each region for each vector. Error bars represent 95% confidence intervals. A one-way ANOVA showed there was a significant effect of retinal region on GFP labelling; $F(2,6) = 14.6$, $p < 0.01$ for AAV2-CAG-GFP and $F(1,8) = 14.0$, $p < 0.01$ for AAV2-DCX-GFP.

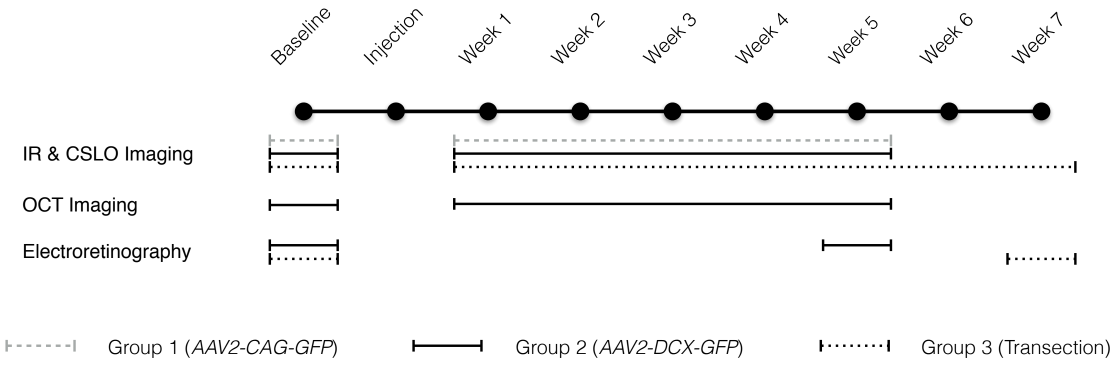

Supplementary Figure S4 Timeline for *in vivo* procedures performed on each group of animals. Imaging (infrared [IR], fluorescence confocal scanning laser ophthalmoscopy (CSLO) and optical coherence tomography [OCT]) and electroretinography were performed repeatedly on animals within the same groups as shown in the timeline. All animals were sacrificed at week 5, except those in the optic nerve transection group that were sacrificed at week 7 (14 days post-transection).

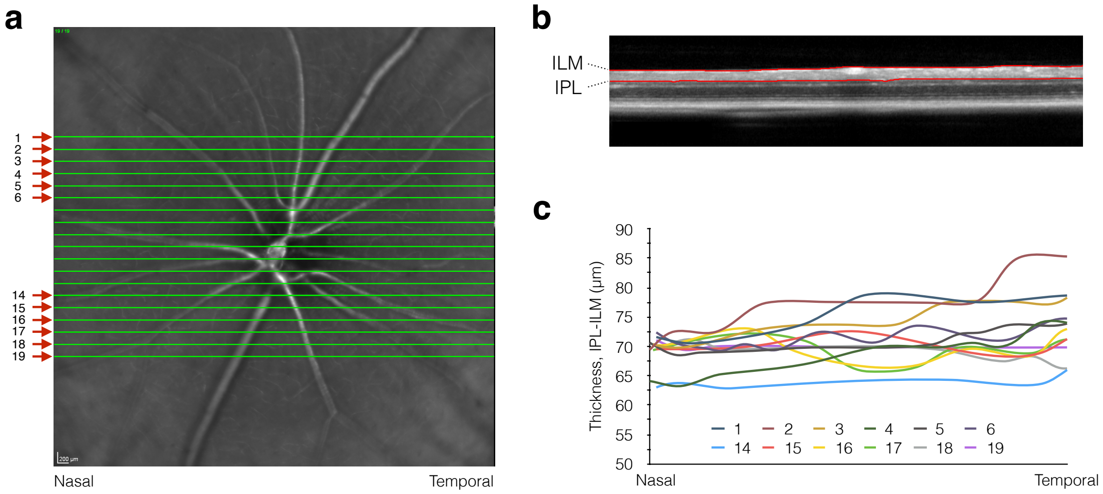

Supplementary Figure S5 OCT imaging and analysis procedures. Optical coherence tomography (OCT) imaging was performed longitudinally in each animal. **a)** Infrared image of a mouse retina centred on the optic nerve head with the raster scanning pattern for OCT (*green lines*). Six B-scans from the top and bottom (total of 12) of the pattern, marked by red arrows, were segmented. **b)** Segmentation of the inner retina (*red lines*) defining the inner limiting membrane (ILM) and inner plexiform layer (IPL). **c)** Thicknesses of the IPL-ILM region plotted for each of the B-scans marked by red arrows in (A). An average thickness was computed for each animal at each time point.
